# “It’s not easy to acknowledge that I’m ill”: a qualitative investigation into the health seeking behavior of rural Palestinian women

**DOI:** 10.1186/1472-6874-13-26

**Published:** 2013-05-24

**Authors:** Linda Majaj, Majed Nassar, Manuela De Allegri

**Affiliations:** 1 UNICEF Bethlehem, West Bank occupied Palestinian territory, Heidelberg, Germany; 2 Medical Aid for Palestinians Ramallah West Bank occupied Palestinian territory, Bethlehem, West Bank; 3 Institute of Public Health, University of Heidelberg, Heidelberg, Germany

**Keywords:** Occupied Palestinian territories (oPt), Women’s health, Health seeking, Andersen model

## Abstract

**Background:**

This qualitative study sets to fill a gap in knowledge by exploring the health seeking behaviour of rural women living in the occupied Palestinian territories (oPt). The existing literature on the oPt has so far focused on unravelling the country’s epidemiological and health system profile, but has largely neglected the assessment of factors shaping people’s decisions on health care use.

**Methods:**

Based on a conceptual framework rooted in the Anderson behavioural model, we conducted 30 semi-structured interviews with purposely selected women and seven key informant interviews in three purposely selected villages in Ramallah district.

**Results:**

Our findings indicate that women delay seeking professional care, use self-prescribed medications and home treatment, and do not use preventive and educational health services. Their health seeking behaviour is the result of the interplay of several factors: their gendered socio-cultural role; their health beliefs; financial affordability and geographical accessibility; their perceptions of the quality of care; and their perceived health needs.

**Conclusions:**

Findings are discussed in the light of their policy implications, suggesting that adequate health policy planning ought to take into considerations socio-cultural dimensions beyond those directly pertinent to the health care system.

## Background

In recent years, several authors have drawn attention to the health conditions of people living in the occupied Palestinian Territories (oPt). They have described the deteriorating living conditions of the Palestinian population, which affect both their well-being and their health status. In addition, they have provided a critical description of the current Palestinian health system, with a focus on maternal health and chronic diseases [1-5].

With a population of approximately four million, Palestinians live in two administratively segregated areas: the West Bank (WB) and the Gaza Strip (GS) [6]. Forty percent of the population is represented by women of reproductive age and children [7]. Although the Palestinian population is quite young (46% under the age of 15), chronic diseases are a major concern, because the country is undergoing a rapid epidemiological transition [3]. Cardiovascular diseases, diabetes mellitus, and cancer are major causes of morbidity and mortality [3]. Moreover, living conditions remain challenging, with high levels of poverty, unemployment, and Israeli blockades restricting movement and livelihood activities, including access to healthcare [2,8].

In 2010, per capita health expenditure from all sources was USD 282, equivalent to 13.7% of GDP [9]. Government and households contributed respectively 36% and 41% of total health expenditure [9]. In turn, given socio-political instability, the ability of the government to fund the health system, is largely dependent on donor funding, which in spite of its magnitude, is still not sufficient to ensure that Palestinians do not also face high out of pocket payments [8,10]. The entire population in the oPt is eligible to receive governmental health insurance (GHI), which is meant to serve as the basis for future social health insurance (SHI) and which grants low cost access to all governmental health care services and to services provided by selected contracted NGOs. GHI covers inpatient and outpatient provider fees, but co-payments apply to medications and to selected specialized health services. Co-payments are generally high considering the country overall socio-economic profile and range from an average of 5.4 USD at governmental facilities to 32.3 USD at NGOs facilities and reaching as high as 43.5 USD at private facilities [10]. Following an emergency measure issued in 2000, over 90% of the population is currently covered by GHI. In addition, about 3% of the population also enjoys private health insurance coverage [8]. The ultra-poor are covered by social welfare insurance and exempted to pay both co-payments and drugs at governmental and/or contracted health facilities.

Given the political and socio-economic dilemmas of the past six decades, the Palestinian healthcare system also faces important challenges in providing adequate services to its population [11]. Health service provision is inadequate, subject to sudden disruption in times of extreme political unrest, and does not respond to people’s needs efficiently [2]. Political and social instability has caused the health care system to evolve in a highly fragmented manner to include four main service providers: the Ministry of Health (MoH), nongovernmental organization (NGOs), the United Nations Relief and Work Agency for Palestinian Refugees (UNRWA) targeting exclusively Palestinian refugees, and private providers. Although coverage indicators are quite good, surprisingly so given mobility barriers due to political unrest, serious concerns remain on the quality of health care service provision in the country [2].

In spite of this recent interest in the country’s epidemiological and health system profile, researchers and policy makers have paid very little attention to the exploration of factors affecting people’s access to and use of health services. With the exception of one quantitative study in the GS [12], no systematic assessment of people’s health seeking behavior has been conducted in the oPt. In particular, no studies have been conducted to explore women’s health seeking behavior, in spite of the fact that the complex Palestinian context yields an amplified effect on them [5]. Not only are women affected by the challenging living conditions described earlier, but they also endure socio-cultural constraints that affect their wellbeing and quality of life [5,7].

With this qualitative study, we aimed to fill this gap in knowledge by exploring what factors shape women’s health seeking behavior in the oPt. In contrast with the only previous study on health seeking behavior by Abu-Mourad et al. [12], we focused exclusively on rural women, not on the population at large, and did not seek to quantify associations between specific factors and health service use. Rooted in the tradition of qualitative research, we rather aimed to unravel how and why the interaction between complex socio-demographic, cultural, and health system factors shapes rural women’s decisions on health seeking behavior.

## Methods

### Conceptual framework

We adopted the Andersen behavioral model as the conceptual framework guiding our exploration of health seeking behavior among rural Palestinian women. Andersen developed the original model in the 1960’s to: 1) understand why families use health services, including both traditional and modern ones; 2) measure equity in access to healthcare; and 3) guide policy-makers in developing equitable health policies. Later, he introduced the individual as the preferred unit of analysis, because of the heterogeneity of family members [13].

**Figure 1:**
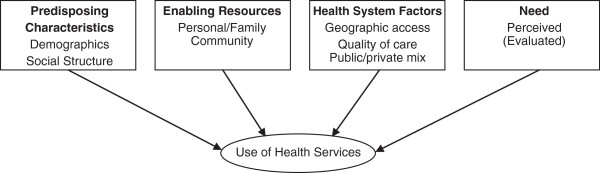
Adapted Andersen model.

Andersen explained the use of health services by individuals as a function of three major components: 1) predisposing characteristics; 2) enabling resources; and 3) need (Figure 1). Predisposing characteristics refer to demography, social structure, and health beliefs and include factors such as: ethnicity, occupation, education, social interactions and networks, values, beliefs, and attitudes. Personal and family enabling resources comprise factors that facilitate the utilization of health services, such as: knowledge of the provided services, income, ability to travel, affordability, and health insurance. Community enabling resources take into account the availability of health services in the community, the presence of a regular source of care, waiting times, quality of care, and users’ satisfaction and perceptions of the provided care. Need factors include both the individual’s perceived health needs, i.e. a subjective evaluation of one’s health status, and the provider’s assessed health needs, i.e. an evaluation of one’s health status based on established medical knowledge and practice.

In a revised version of the model, Andersen argued that single researchers could add more factors to the original model, without disrupting its original structure, to fit the purpose and nature of their research. Hence, we adopted a revised version of Andersen’s model. Based on the criticism of the original model, this revised version allowed for the inclusion of one additional factor which we deemed to be important in the Palestinian context, i.e. the organizational structure of health service provision [13].

### Study design

Unlike many prior studies which rely on Andersen’s behavioral model as the guiding framework for the analysis of health seeking behavior [14-16], we chose to rely on qualitative rather than quantitative methods. Our choice was motivated by several factors. First, the paucity of evidence on the matter made it imperative that we defined the study as exploratory, i.e. as an initial attempt to understand what factors play a role in women’s decision on health seeking in the oPt, and the literature clearly identifies qualitative methods as better suited for exploration of new areas of knowledge than quantitative ones [17,18]. Second, our wish was to explore health seeking behavior in a holistic manner to understand how decisions are made and why, rather than quantitatively distilling the association between a handful of independent variables on a limited set of selected outcomes [19,20]. Moreover, in the absence of sufficient prior knowledge on women’s health seeking in the oPt, it would have been close to impossible to even define appropriate outcomes.

### Study setting

We conducted the study between May and July 2010 in the WB. As specific study site, we purposely selected three villages in the Ramallah district, which we judged to be representative of the WB rural context, reflected different levels of progressive urbanization, and could be reached relatively easily by the study team. With an average of 4000 inhabitants per village, there were numerous community facilities in each village. These included: schools and kindergartens, community centers, and sport facilities. In addition to agriculture, the main sources of income were small local businesses and waged labor. Women in the villages were mostly housewives and sometimes helped informally with family businesses and agriculture. Only a minority of women were employed and worked in the formal sector either in the villages or in neighboring cities.

Like in most oPt rural areas, primary healthcare services were provided in decentralized health centers run by various NGOs and/or by the MoH. Private general physicians also provided primary healthcare services. The services available included curative, preventive, and educational services for women and children, including general and specialized medicine for acute and chronic diseases, and dentistry. With the exception of the services provided by individual private physicians, most health services were partially or fully subsidized by the government or by donor agencies at the general conditions described earlier in the introduction. Secondary healthcare services were absent in the villages, but provided at the district hospital and in private hospitals located in the district capital. On average, these villages were 15 to 20 km away from the district capital, but patients often had to travel 30 to 40 km because of frequent Israeli road blocks. People traveled with private means or with public transportation, but in case of emergency, ambulances were also available in the villages.

### Sample selection

We conducted 30 individual semi-structured interviews with women (10 per village). In line with prior research [21-24], we privileged individual interviews above focus group discussions, because we wished to focus on individual experiences, having the possibility to address sensitive matters in a confidential setting [18,25], to fully comprehend the complexity of the decision making process shaping women’s health seeking. We purposely selected the women to interview on the basis of their educational and working status, since we assumed, in line with available evidence from other settings [26-29], that these two traits could be fundamental in shaping women’s decision [18]. In each of the selected villages, we identified five women who were educated (minimum three years college education) and worked and five women who had only basic or no schooling and were not engaged in the formal economy. This heterogeneous sampling technique aimed to develop an analysis which allowed for the exploration of variations in experiences because of women’s background, the rationale being that any common pattern emerging from such variation would provide powerful insight into the research question [30]. We were ready to interview more women if needed, but since saturation and redundancy were amply reached with the initial sample, this was not necessary.

The first author contacted the women to interview either at community gatherings (non-working women) or at their working places (working women). On these occasions, the first author identified women who fulfilled the selection criteria, explained the aim of the study, and asked for permission to set an appointment for a later interview. Women could freely refuse the interview. Three women declined to be interviewed, thus we contacted a total of 33 women to conduct 30 interviews.

In addition, to triangulate the information provided by the women [17,18] and to contextualize findings in a more comprehensive manner, we conducted seven in-depth interviews with key informants. These informants were: 1) the community representative in each village council or municipality (n = 3); 2) the medical director of the health centre in each village (n = 3); and 3) the director of the Mother and Child Health Program for the Ramallah district at the Ministry of Health (n = 1).

### Data collection

We used two different interview guides: one for the women and one for the key informants. The first author developed the interview guides in English with the assistance of the second and the third author to reflect the conceptual framework described in Figure 1. In both guides, we addressed the same set of issues, but from different perspectives. In the guide used to interview women, we focused on the individual experience of health seeking. In the guide used to interview key informants, we explored the institutional perspective on women’s health seeking. In addition, we used the interviews with key informants to contextualize women’s findings, by gathering information on health care provision in the area. Prior to the beginning of the actual data collection, we piloted the interview guides on one woman in each of the selected villages. We made amendments to the guides as a result of these pilot interviews.

The first author, who is herself a Palestinian woman, translated all interview guides into Arabic and conducted all interviews in colloquial Arabic. Interviews lasted on average 30 to 45 minutes. To ensure privacy, we interviewed respondents alone in their own houses or at their workplace. Before proceeding with the interview, the first author explained again the aim of the study and sough informed consent to participate. We tape-recorded all interviews. In addition, the first author noted the core of the answers while she proceeded through each interview.

### Data analysis

The first author transcribed and translated all interviews into English and matched the transcripts with the notes and memos she had taken during the data collection process [31]. Initially, the first author coded the transcribed text manually, using a deductive coding scheme which reflected the categories of the conceptual framework and of topics addressed in the interview guide [18,32]. To take into account unexpected themes which emerged during the course of the interviews, the first author created additional codes while she proceeded through the reading. Then, she reduced the coded material into categories and sub-categories. The third author also independently classified the coded material to illuminate understanding of the research question and to triangulate the first author’s analysis. As the final stage of the analysis, all three authors worked together to identify links between categories, to triangulate the information reported by the women with those reported by the key informants, and to interpret the emerging findings in the light of their knowledge of the situation in the oPt.

## Results

We have organized the presentation of the results in two sections: a description of the women and of their health seeking patterns and factors explaining women’s health seeking patterns. We illustrate findings using verbatim quotations, translated into English by the first author.

### General description of the health seeking trends

Women’s age ranged between 25 and 52 years, with a median of 34.5 years. Most women were married; three were either single or divorced. The average family size was 6.75 persons, with an average of five children per woman. Like the majority of the Palestinian population, all women were Arab and Muslim. The educational level of the respondents varied considerably. The spectrum ranged from obtaining higher university degrees to no schooling at all. When asked to evaluate their socio- economic status, most women identified themselves as belonging to the middle-income group. Two women indicated being well off and three indicated being extremely poor.

All working women had GHI through their job, granting coverage for health service use at governmental facilities for themselves and their dependents. With the exception of one woman, non-working women also had GHI, either through their husbands’ job, or because they had applied for the free emergency coverage issued in 2000 [8]. Only two women had private health insurance.

The vast majority of women rated their health status as good. On one hand, women reported a certain delay in seeking professional care when ill. They explained that for common conditions, such as urinary tract infections, fatigue, and flu, they first resorted to self-care, including the use of traditional herbal remedies. The traditional remedies most frequently mentioned were: olive oil for tonsillitis, parsley for urinary tract infections, herbal teas (chamomile, thyme, and sage) and garlic for the common cold and the flu. Women sought formal care only for conditions that did not improve with self-care or for conditions that were at once perceived to be severe, such as injuries, cardiovascular diseases, and diabetes. On another hand, women reported seeking prompt medical assistance when pregnant or for delivery. All women had attended at least nine anti-natal care (ANC) visits per pregnancy and had delivered in the presence of a skilled professional attendant. Women seldom utilized preventive, including screening, and educational health services, in spite of their wide availability in the villages. Key informants confirmed that a wide range of preventive services, including pap smears, mammography, regular blood tests (for diabetes, hyper lipidemia, osteoporosis), and educational sessions, including home visits by community health workers, were available at the village level, but remained under-utilized.

Women expressed a generalized preference for private health care providers. They explained, however, that their choice to use public rather than private services was often mediated by a series of social, economic, and geographical considerations.

### Factors affecting women’s health seeking behavior

#### Perceived need and predisposing factors

When describing their health status, all women cited “*Alhamdulillah”*, an Arabic expression which indicates “Thank God”, even if they suffered from various ailments. Their statements revealed that rather than articulating a sincere judgment on their health status, they responded to the social expectation not to discuss publicly their illnesses: *“Although I have several illnesses I cannot complain, all I can say is Alhamdulillah!”* (village 1, non-working, non-educated, 52 years old). Educated working women, however, discussed their health concerns more openly and expressed higher awareness of their health needs: *“We [my husband and I] are both well-educated and are aware of our health needs”* (village 2, working, educated, 36 years old). They were better able to identify causes and reasons to suffer from ill health which were aligned with modern medical knowledge. Thus, they could recognize in the services available in their community the logical opportunity to restore their health, irrespective of whether they always took such opportunity. Key informants confirmed that less educated women had greater difficulties in identifying their health needs, especially in relation to conditions that did not affect them in the immediate present.

When discussing the use of health care services in relation to their health needs, women consistently asserted that they were first and foremost housewives and caretakers of their children and families. They explained their preference for self-care in relation to the little time and opportunity available to look after their own health: *“It’s true that I am a working woman, but at the end of the day I am first a mother and a wife. Having all these roles as a woman leaves little time to look after my own wellbeing”* (village 3, working, educated, 29 years old). The preference for self-care was observed among women of all educational and socio-economic statuses, suggesting that women’s responsibility toward the family was a stronger element in mediating women’s decision on curative health care seeking than their education. Lack of time was also used to explain why women took little advantage of the educational and preventive services available in the villages.

Key informants felt that women had greater health needs than what they expressed by their health care use. They indirectly linked women’s limited use of professional health care services to low education. They explained that because of early marriage, many women leave school before completing it. In addition, they indicated that early marriage translates into a high number of pregnancies, often complicated ones because of women’s young age, leaving women with little chance to look after their own health needs once they have cared for those of several children.

*“I have seven children and I was married at a young age…I suffer from constant fatigue, I am anemic …but there is not much that I can do about it… if I need to go see the doctor, there is no one to take care of the children, as my husband is at his work all day long”.* (village 1, non-working, non-educated, 40 years old)

*“We have been stressing the importance of women’s education and awareness raising. Girls marry at a very young age here… It takes a lot of effort, but we try to support the local community and women’s education as much we can. That’s the only way to address behavioral change and improve women’s use of health care services”.* (village 3, medical doctor)

The majority of women also justified their delay in seeking professional care when ill in relation to the socio-cultural norms prevalent in the villages. They felt that they were not expected to discuss their illness, to express any complaint about it, or to visit a doctor at the first onset of symptoms, without having first attempted to solve the problem on their own: *“It is not easy to acknowledge that I am ill. I take herbal medications at home when I feel ill. If I do not get better then I go to the see the doctor”* (village 2, working, educated, 32 years old). Trust in the effectiveness of herbal remedies induced women to frequently prefer them to modern medications as first-line treatment. In addition, women attributed to their knowledge and experience with the most common diseases, such as the seasonal flu and urinary tract infections, their ability to use over-the-counter drugs and herbal remedies as first-line treatment.

Women believed that health matters are in the hands of Allah: *“Of course screening for cancer is important and I might consider doing mammography and a pap smear, but it won’t change the fact that health and illness are in the hands of Allah”* (village 2, working, educated, 43 years old). This belief was deeply rooted among women of all educational and socio-economic statuses. Trust in Allah made women less worried of their ill health and made many health services, especially educational and preventive ones, appear of secondary importance. Adding to this, fear to discover a cancer, uneasiness with invasive screening procedures, and women’s lack of reciprocal encouragement further limited the use of preventive services among women of all socio-economic groups: *“Prevention is not a culture in rural society; we do not go see a doctor until we fall very ill”* (village 2, non-working, non- educated, 39 years old).

#### Enabling factors

Most women indicated that their families supported them when in need for medical care. Husbands, children, and in-laws, all encouraged women to seek care and helped out in case of illness. Family support, however, was again bound to the differentiation between minor illnesses which could be treated at home and severe ones which needed professional attention. Obtaining husband’s consent was crucial to seeking professional care, especially for the non-working women: *“My husband encourages me to seek professional care when he feels it is serious illness. When not, he tells me to take something at home”* (village 1, working, educated 27 years old).

All interviewed women were aware of the various providers and services available in the villages. Women identified the quality of the services on offer as an essential element in shaping their health seeking decisions. Quality of care was defined by most women in terms of respectful provider-patient interactions, shorter waiting times, and good reputation in the community. All women agreed that private providers offered higher quality services than governmental facilities: *“I hear that they* [staff at governmental hospitals] *shout at women and are rude to them…… At private hospitals, women are treated with more respect than at governmental ones”* (village 3, working, educated, 35 years old)*.* Women’s provider choice for curative care, however, was often mediated by considerations of affordability. Working women could afford to use services offered by private providers. Non-working women and women of lower socio-economic status tended to use governmental services, because they were more affordable. In many instances, irrespective of one’s education and socio-economic status, women wished to resort to private providers for maternal care services, even if they had to pay for them: *“I do not mind paying out of pocket for maternal services, as long as I receive good care”* (village 2, working, educated, 46 years old). For chronic diseases and surgeries, governmental services were indicated as the only option for most women, because of coverage by GHI: “*When it comes to more expensive services, women choose those offered at the governmental health facilities, as people here need to look for cheaper or free of charge services*” (village 2, medical doctor).

Considerations of geographical accessibility also mediated women’s choice for health care providers. In spite of the political conflict which obstructs movement between the different parts of the WB, key informants reported that referral across levels of care was well functioning and facilitated by a fairly good public transportation system. Women, however, complained of the lack of availability of secondary health care services in the villages, because they perceived distance and the time needed to travel to the main cities as additional obstacles to access given their busy lives. Women of higher socio-economic status had easier access to private transportation means and were thus more inclined to use services in the main cities.

*“It is quite difficult to access services outside the village. Here everything is within walking distance. Going to the nearest city [Ramallah] takes at a long time … with the presence of Israeli checkpoints and going through bypass roads, it becomes even harder and expensive for people with a limited income like us”.* (village 1, non-working, non-educated, 51 years old)

*“Services here in the village are quite accessible to women. The health center is within walking distance from most houses. When services are needed that are not available in the village, we coordinate with the local community ambulance to transport the patients in case of emergency. In normal cases affordable public transportation is always available at the village”.* (village 1, medical doctor and community representative)

## Discussion

### Methodological considerations

With this study, we have set the first attempt to use qualitative methods to explore women’s health seeking behavior in the oPt. In doing so, we have complemented existing literature on the epidemiological profile of the nation [5,33], on the functioning of the local health system [4,8,34], and on the very limited evidence on determinants of access to care generated from quantitative studies [12,35]. The use of the Anderson model [13] as the basis for the conceptual framework guiding data collection and analysis made our study potentially replicable in other settings and allowed for a more transparent comparison with findings emerging from other studies. The fact that the first author was herself a Palestinian woman, assisted on the field by another Palestinian (i.e. the second author), greatly facilitated data collection, ensuring that the interviews could be conducted in a culturally sensitive manner. The fact that someone internal to the society (i.e. the first author) and by someone external to the society (i.e. the third author) worked together on the analysis enhanced the triangulation process, often forcing the authors to question the material again in the light of discordant emerging interpretations [30].

In spite of the relatively small number of women interviewed, we are confident that redundancy and saturation were reached because no new concepts emerged in the coding of the last six to eight interviews. The limited number of villages selected, however, does pose a challenge to the transferability of the results to the wider WB context. The selection of the villages in fact, was also shaped by pragmatic considerations on accessibility because of the political conflict and to the frequent road blockages. While we are confident that we could have not done any better given the circumstances under which the study was conducted, we are aware that women living in very remote areas of the WB might experience more extreme conditions than those shared by women in our study. Thus, our findings should be read as representing a “lower bound estimate” of what the socio-cultural, geographical, and health system factors that shape women’s health care seeking in the oPt.

### Policy implications

With our study, we showed that women often resorted to self-treatment, delayed seeking professional care, and under-used educational and preventive health services. In line with Andersen’s model [13], we revealed that women’s choices regarding health seeking were shaped by a combination of predisposing, enabling, and structural factors and perceived need. In particular, we identified socio-cultural influences as the central element shaping women’s health seeking behavior. Specifically, women’s gendered role within the family and in society as whole, Arab socio-cultural norms, and women’s health beliefs were found to be key in shaping women’s choices regarding their health. Elements related to the organizational structure of the health system, including quality of care, accessibility, and affordability, were also found to affect women’s health seeking. Women barely mentioned the prevailing political conflict as an element shaping their health seeking decisions, although they recognized its influence on impaired mobility and deteriorating living conditions.

This absence of focus on the political conflict in women’s discourse may at first sight appear surprising. The reader, however, must bear in mind that interviews were conducted in an open manner, with no explicit focus on the political conflict with Israel. Respondents could raise the point, if they wished to do so, but were not repeatedly probed to do so. The authors purposely selected this strategy, because they felt that all that is disseminated and therefore known on the oPt revolves around the conflict, with little attention being paid to other dimension of everyday life. Women did not insisted on the role of the conflict in mediating their health seeking decisions in an explicit manner, but rather allowed it to appear in between the lines when recognizing the limitations of their health care system. This is probably an indication that they are accustomed to the situation, potentially having lived their entire life with the conflict in the background, to the extent that they no longer recognize its explicit role in shaping their health seeking decisions. The most likely interpretation is that socio-political unrest molded and continues to mold the features of the health care system, as described in the introduction, but not necessarily people’s everyday decisions on health seeking, at least not on a conscious level.

Women’s evaluation of their health status and their perceived need for care represent the first indication of the socio-cultural influences acting on their health seeking. Women in fact, tended to rate their health status positively and to understate the need for care even in the presence of disease. This tendency, which has been observed before in Arab societies as well as among Arab women living in non-Arab societies [16,17], has been attributed to deeply internalized social and cultural norms, which induce women to conform to the expectation to be healthy and not to express publicly any complain on their health. Awareness of such behavior holds important consequences for health care provision. Health care providers should take into consideration women’s tendency to understate their health complaints and work to evaluate women’s objective health needs in relation to women’s subjective perceptions [36]. Interventions should be designed to help women move beyond cultural expectations by recognizing and expressing health needs more openly.

Similarly to what previously reported by other authors in a variety of other settings [37-40], this internalized expectation to be healthy inevitably limited women’s use of health services, in particular preventive ones, such as cancer screening. Women indicated that their use of health services was further limited by their gendered role within the family and a traditional social structure. Like in many other Arab countries, most rural women in the WB are bound to early marriage, repeated childbearing, and household responsibilities [5,41,42]. Irrespective of socio-economic and educational status, these factors encumbered on women’s time and on their chance to care for their own health needs. Furthermore, in spite of being more aware of their health needs, even educated women could often not act upon them because of the socio-cultural constraints discussed so far.

Hence we postulated that, similarly to what observed in other settings [39,40], women’s education on its own is not enough to overcome socio-cultural influences on women’s health seeking behavior. Additional strategies, such as complex national political and economic interventions, ought to be developed to foster women’s empowerment, thus enabling women to overcome socio-cultural barriers in access to care [43,44]. The oPt could learn from the experience of other countries where working to enhance women’s economic autonomy and to foster women networks, for instance through the provision of micro-credit programs, resulted in overall empowerment and ultimately in a more informed use of health care services [45-49].

Confirming findings from previous studies conducted among Arab women in other settings [38,39,50], we suggested that women’s use of health services, especially with regard to prevention and care for chronic diseases, was further limited by the deeply rooted belief that health is in the hands of Allah. In line with previous theoretical as well as empirical research [51,52], this reinforced the principle that risk perceptions are embedded in cultural, ethical and religious traditions.

In the light of the increasing spread of chronic diseases [6], such belief represents a major challenge for policy makers and health providers, especially in relation to the provision of preventive care services, including early cancer screening [53]. Some authors have further postulated that fear to contradict such dominant religious beliefs might also induce Arab women not to utilize preventive care services out of fear of facing stigma in their community [37,54]. Involving religious and community leaders to discern health risks from religious beliefs represents an essential gateway to increase women’s awareness and encourage them to use preventive health services, in particular cancer screening [55].

The influence of culturally rooted health beliefs on women’s health seeking behavior is also reflected in women’s extensive use of herbal remedies as an alternative to professional care. Other authors also reported similar findings in a previous study among female university students in the WB [56], confirming a pattern which appears to be common both in many other low and middle income countries and among certain ethnic groups in high income countries [57-59]. The co-existence of folk [home], traditional, and modern medicine is actually a well-documented phenomena across the world and does by no means appear surprising [60]. This utilization pattern, however, raises important questions when considered in relation to the overall affordability of formal health services, including both direct monetary costs and time costs of consuming care. Similarly to what observed in other settings [58,61], women’s use of informal care in fact, was not motivated by mere preference, but more importantly by overall considerations on the accessibility of formal health care services. Furthermore, women’s use of informal care, including both over the counter and traditional drugs, raises important concerns on treatment efficacy, potential herb-drug and drug-drug interactions, and the development of resistance [62-66]. Health policy makers should be aware of the complexity of women’s utilization patterns and address the issue with effective health education interventions, aimed at informing, if not reducing, the use of informal care.

Women also cited poor quality of care as the primary reason not to prefer governmental health services above private ones in spite of their lower price. Women’s willingness to pay for better quality at private facilities corroborates existing evidence on the poor quality of health care services at public facilities in the oPt, especially maternal care services [2,5,67,68] and reflects findings from other settings [69-72]. However, while women in our study were primarily concerned with process indicators of health service delivery, such as waiting times and providers’ attitudes, previous research had focused on the assessment on system inputs, primarily the existence of an insufficient and poorly trained health workforce to meet the countries needs [73].

Jointly assessing evidence from our study and from previous studies [74,75] suggests that investments in quality are likely to result in increased health service utilization and possibly in a shift from private to public providers. Similar to the experience of other middle income countries [34,76-79], public-private partnerships could also contribute to improve access to quality care. Such partnerships can probably be developed effectively only in a context of improved risk-pooling across the population [4], expanding current health insurance coverage on all three dimensions [i.e. number of people covered, proportion of costs covered, and number of services covered] indicated in the recent World Health Report [80]. Women’s use of services upon the payment of fees testifies the existence of a certain ability to pay which could be better capitalized if channeled toward fostering the expansion of insurance coverage.

## Conclusions

In conclusion, with this study, we indicated that a complexity of factors, i.e. socio-cultural norms and values, geographical and financial accessibility, and health system structures, including quality of care, shape women’s decisions on health care seeking in the oPt. Together with existing evidence on the changing epidemiological profile of the nation [6], we provide the necessary evidence-base for the development of effective and responsive health care policies in the country, even in the presence of difficult socio-political circumstances.

### Ethical clearance

This study was exempted from ethical approval by Palestine National Authority at the time of conducting the study. Ethical clearance was granted by Ministry of Health of Palestine.

## Competing interests

We declare that we have no conflicts of interest.

## Authors’ contribution

LM and MDA were responsible for the study design. LM prepared and translated the data collection tools with contributions from MDA and MN. LM, with support from MN, was responsible for data collection. LM and MDA did the data analysis. All authors contributed to the literature review and to writing the manuscript. All authors read and approved the final manuscript.

## Pre-publication history

The pre-publication history for this paper can be accessed here:

http://www.biomedcentral.com/1472-6874/13/26/prepub
